# Intra-operative cell salvage in radical cystectomy

**DOI:** 10.4103/0970-1591.65379

**Published:** 2010

**Authors:** Abhay Rane

**Affiliations:** Department of Urology, Surrey and Sussex NHS Trust, United Kingdom. E-mail: a.rane@btinternet.com

Perioperative allogeneic blood transfusion is regularly utilized in major pelvic uro-oncological surgery, although the requirements have decreased over the past few years, primarily due to improved technical maneuvers and the availability and uptake of laparoscopic / robotic routes. However, wherever the risk is quantifiable, it makes sound medical sense to utilize the cell saver, with its lower risk of transfusion related reactions. The authors[1] have elegantly demonstrated a significant monetary advantage as well, with cell salvage, in 15 consecutive patients undergoing radical cystectomy.

In general, the trend appears to be one of opting for use of cell salvage wherever possible, primarily due to the shortage of easily available allogeneic blood, and the possibility of transfusion related reactions. One of the concerns raised by many oncologic surgeons relates to the theoretical risk of dissemination of cancer. However, studies by various authors indicate that the survival rate of patients who received cell salvaged blood did not significantly differ from the group of patients who did not receive cell salvaged blood. These studies have been referenced in the article.

A number of metanalyses[2] have confirmed that cell salvage may be a cost-effective method to reduce exposure to allogeneic blood transfusion in all fields of surgery. Cell salvage has lower costs and slightly higher quality-adjusted life years compared with all of the alternative transfusion strategies except for acute normovolaemic haemodilution, which has not been in routine practice for oncological surgery.
